# Development and validation of a nomogram to predict drainage duration in patients with breast cancer treated with modified radical mastectomy

**DOI:** 10.1038/s41598-021-82073-y

**Published:** 2021-01-28

**Authors:** Song Wu, Zechang Xin, Daxing Sui, Zhengli Ou, Haotian Bai, Shenzhen Zhu, Xueying Wang, Jiaxin Zhang

**Affiliations:** 1 Department of Thyroid and Breast Surgery, The First People’s Hospital of Wenling, Wenling, Zhejiang China; 2 Dalian Medical University, Dalian, Liaoning China; 3 Department of Thyroid and Breast Surgery, Yangzhou University Affiliated Northern Jiangsu People’s Hospital, Yangzhou University Medical Academy, Yangzhou, 225001 Jiangsu China

**Keywords:** Breast cancer, Risk factors

## Abstract

Appropriate drainage duration is vital for the postoperative rehabilitation of patients with breast cancer (BC) undergoing modified radical mastectomy (MRM). To provide better and individualized postoperative management for these patients, this study explored independent predictors of postoperative drainage duration in patients with BC. This was a single-center retrospective cohort study. Patients diagnosed with BC and treated with MRM from May 2016 to April 2020 were randomly divided into training (n = 729) and validation (n = 243) cohorts. Univariate and multivariate Cox analyses revealed that the body mass index, serum albumin level, hypertension, number of total dissected axillary lymph nodes, and ratio of positive axillary lymph nodes were independent predictors of postoperative drainage duration in the training cohort. Based on independent predictors, a nomogram was constructed to predict the median postoperative drainage duration and the probability of retaining the suction drain during this period. This nomogram had good concordance and discrimination both in the training and validation cohorts and could effectively predict the probability of retaining the suction drain during drainage, thus assisting clinicians in predicting postoperative drainage duration and providing individualized postoperative management for patients with BC.

## Introduction

Breast cancer (BC) is a critical public health threat to women worldwide^1^. At present, the treatment of BC mainly includes surgical operation, radiotherapy, chemotherapy, endocrine therapy, and targeted therapy^2^. Modified radical mastectomy (MRM) is a widely used surgical method for invasive BC; it is also the gold standard for the staging of axillary lymph nodes in patients with axillary lymph node metastasis^3^. Patients undergoing MRM usually have varied postoperative drainage durations due to the large surgical wound and the injury to blood vessels and lymphatic vessels during the surgery^4^. No consensus exists on the timing of suction drain removal. However, the premature removal of the suction drain may lead to seroma formation, thus increasing the risk of infection, arm lymphedema, and flap necrosis. In addition, later extubation limits postoperative shoulder exercise and prolongs the hospital stay of patients^5^.

Hypertension and obesity were recognized as factors that prolong drainage duration in patients undergoing mastectomy and axillary dissection^6–10^. A few other predictors have also been reported, but no model was available for predicting postoperative drainage duration. Therefore, exploring more predictors and constructing a predictive model of drainage duration was necessary so as to provide better and individualized postoperative management for patients. Nomogram is a convenient and intuitive modeling tool that can quantify demographic and clinical variables to generate a visible model that can predict a specific event^11^.

The purpose of this study was to explore factors related to drainage duration in patients undergoing MRM. Based on this, an efficient model was explored, and a nomogram was constructed that could be used for an individualized assessment of drainage duration in patients with BC.

## Materials and methods

### Patients selection and data acquisition

This study involved female patients with unilateral BC who underwent MRM at Northern Jiangsu People’s Hospital between May 2016 and April 2020. During the surgery, an ultrasonic scalpel was used to dissect axillary lymph nodes, and larger lymphatic vessels were ligated using absorbable sutures. After mastectomy and axillary dissection, two suction drains were placed to provide negative pressure suction: one at the axilla and the other at the chest wall. Besides, elastic bandage and external compression dressing were used to obliterate the dead space of the chest wall and axilla after the surgery. The exclusion criteria were as follows: (1) the number of total dissected axillary lymph nodes less than 10, (2) received neoadjuvant therapy before the surgery, (3) distant metastases occurred when patients were diagnosed, (4) drainage duration of axillary suction drain more than 3 weeks, (5) patients with seroma formation after removing axillary suction drain, (6) patients with an incomplete record of drainage duration, (7) patients with a disease that may change hematological data, and (8) patients with other complications. Finally, 972 patients were included in the study.

The following data were collected for each patient: patient ID, age, laterality of tumor, body mass index (BMI), peripheral blood lymphocyte count (PBLC), serum albumin level, hypertension, diabetes, type of intraoperative fluid, use of painkillers after surgery, number of positive axillary lymph nodes (PLNs), number of total dissected axillary lymph nodes (TLNs), drainage duration of chest wall suction drain (DDCSD), and drainage duration of axillary suction drain (DDASD). The drainage fluid was emptied and measured every 24 h. The criterion of chest wall and axillary suction drain removal was total drain output of 20 mL or less daily for 2 days starting from postoperative day 3. The chest wall suction drain was removed before the axillary suction drain.

The need for informed consent was waived by approving ethics committee because of the retrospective nature of the study, and the study design was approved by the Medical Ethics Committee of Northern Jiangsu People's Hospital.

### Independent predictors of DDASD

The eligible patients were randomly divided into training cohort and validation cohort. The training cohort was used to explore independent predictors of DDASD and construct a predictive nomogram model. First, univariate Cox models were employed to identify the relationships between clinicopathological characteristics and DDASD of patients. Variables significantly correlated with the DDASD were screened out by the *P* value < 0.05 in the univariate Cox model. Second, these significant variables were further fitted into a multivariate Cox model. The variance inflation factor (VIF) value of each variable was checked to confirm the collinearity among variables. Finally, variables with the *P* value < 0.05 in the multivariate Cox model were considered as independent predictors and used to construct the nomogram model.

### Construction and validation of the nomogram

A predictive nomogram model was constructed based on the results of the multivariate Cox model. This nomogram was validated using the validation cohort and internally processed. The concordance index (C-index) with a 95% confidence interval (95% CI) was calculated in the training and validation cohorts to evaluate the discrimination of the nomogram. Calibration plots (500 bootstrap resamples) were established to check the concordance between the nomogram‐predicted and the actual probability of retaining the suction drain at the median DDASD. Furthermore, receiver operating characteristic (ROC) curves were generated to assess the sensitivity and specificity of the nomogram-predicted probability of retaining the suction drain. The area under the curve (AUC) value with a 95% CI was also calculated.

### Statistical analyses

All statistical analyses were conducted using R software version 4.0.0^12^. Two-sided *P* values < 0.05 were considered statistically significant.

### Ethical approval

This article is a retrospective review of patient data, and the study design was approved by the Medical Ethics Committee of Northern Jiangsu People's Hospital. This article does not contain any studies with animals or human participants performed by any of the authors. All methods were carried out in accordance with relevant guidelines and regulations.

### Informed consent

For this type of study formal consent is not required, and the need for informed consent was waived by approving ethics committee.

## Results

### Clinicopathological characteristics of patients

As shown in Table 1, 729 patients were included in the training cohort and 243 in the validation cohort. The median DDCSD and median DDASD of all patients were 5 days (ranging 4–8 days) and 11 days (ranging 5–21 days), respectively. No statistically significant difference was found between the training and validation cohorts.

**Table 1:** Clinicopathological characteristics of patients with breast cancer in the present study.

Clinicopathological characteristics	All patients	Training cohort	Validation cohort	*P* value
	*n* = 972	*n* = 729	*n* = 243	
**Age, year**				0.191
≤ 45	172 (17.7%)	134 (18.38%)	38 (15.64%)	
> 45, ≤ 55	424 (43.62%)	320 (43.9%)	104 (42.8%)	
> 55, ≤ 65	245 (25.21%)	172 (23.59%)	73 (30.04%)	
> 65	131 (13.48%)	103 (14.13%)	28 (11.52%)	
**Laterality**				0.335
Left	516 (53.09%)	380 (52.13%)	136 (55.97%)	
Right	456 (46.91%)	349 (47.87%)	107 (44.03%)	
BMI (kg/m^2^)^a^	24.13 (22.14–26.19)	24.03 (22.21–26.14)	24.26 (21.92–26.44)	0.715
PBLC (×10^9^/L)^a^	1.61 (1.3–2.03)	1.61 (1.3–2.02)	1.63 (1.32–2.05)	0.349
Albumin (g/L)^a^	48.1 (46.4–50)	48 (46.2–50)	48.3 (46.7–49.9)	0.295
**Hypertension**				0.134
No	812 (83.54%)	601 (82.44%)	211 (86.83%)	
Yes	160 (16.46%)	128 (17.56%)	32 (13.17%)	
**Diabetes**				0.167
No	858 (88.27%)	637 (87.38%)	221 (90.95%)	
Yes	114 (11.73%)	92 (12.62%)	22 (9.05%)	
**Type of intraoperative fluid**				0.531
Crystalloid	261 (26.85%)	200 (27.43%)	61 (25.1%)	
Crystalloid and colloid	711 (73.15%)	529 (72.57%)	182 (74.9%)	
**Use of painkillers after surgery**				0.311
No	931 (95.78%)	695 (95.34%)	236 (97.12%)	
Yes	41 (4.22%)	34 (4.66%)	7 (2.88%)	
PLNs^a^	1 (0–4)	1 (0–4)	1 (0–4)	0.962
TLNs^a^	17 (14–21)	17 (14–21)	17 (14–22)	0.732
**Ratio of PLNs**				0.303
0%	468 (48.15%)	349 (47.87%)	119 (48.97%)	
> 0%, ≤ 15%	203 (20.88%)	151 (20.71%)	52 (21.4%)	
> 15%, ≤ 30%	107 (11.01%)	88 (12.07%)	19 (7.82%)	
> 30%	194 (19.96%)	141 (19.34%)	53 (21.81%)	
DDCSD (d)^b^	5 [4–8]	5 [4–8]	5 [4–8]	0.478
DDASD (d)^b^	11 [5–21]	11 [5–21]	10 [5–21]	0.677

BMI, body mass index; DDASD, drainage duration of axillary suction drain; DDCSD, drainage duration of chest wall suction drain; PBLC, peripheral blood lymphocyte count; PLNs, number of positive axillary lymph nodes; ratio of PLNs, ratio of positive axillary lymph nodes; TLNs, number of total dissected axillary lymph nodes.

^a^Shown as median (interquartile range).*

^b^Shown as median [range].

### Screening for independent predictors of DDASD and developing the nomogram

**Figure 1:**
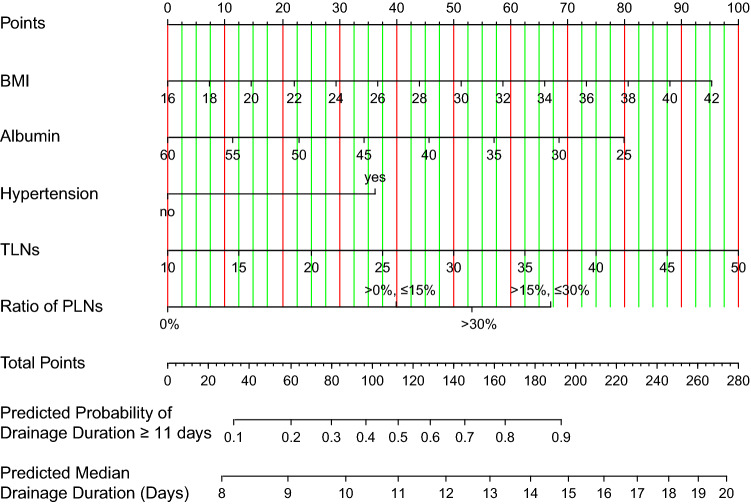
Nomogram to predict the median drainage duration of axillary suction drain and the probability of retaining the suction drain during drainage. BMI, body mass index; PLNs, positive axillary lymph nodes; TLNs, number of total dissected axillary lymph nodes.

The results of univariate and multivariate Cox analyses in the training cohort are shown in Table 2. The univariate analysis showed that BMI, hypertension, TLNs, ratio of PLNs, PLNs, albumin level, and age correlated with the DDASD (*P* < 0.05). These variables were fitted into a multivariate Cox model. The VIF values of all variables were less than 2, indicating no collinearity among variables. BMI, albumin, hypertension, TLNs, and ratio of PLNs were independent predictors of DDASD. Furthermore, hypertension led to a longer DDASD (hazard ratio < 1). The increases in BMI, TLNs, and ratio of PLNs were associated with a longer DDASD (hazard ratio < 1). The increase in the albumin level was associated with a shorter DDASD (hazard ratio > 1). A nomogram was constructed based on the results of the multivariate Cox model to predict the probability of DDASD equal to or more than 11 days and median DDASD (Fig. 1).

**Table 2:** Univariate and multivariate Cox regression analysis of factors affecting axillary drainage duration in patients with breast cancer after modified radical mastectomy.

Variables	Univariate Cox analysis		Multivariate Cox analysis	
	HR (95% CI)	*P* value	HR (95% CI)	*P* value
BMI	0.936 (0.914–0.958)	< 0.001	0.944 (0.921–0.967)	< 0.001
**Hypertension**				
No	Reference		Reference	
Yes	0.641 (0.529–0.777)	< 0.001	0.594 (0.483–0.73)	< 0.001
TLNs	0.966 (0.953–0.979)	< 0.001	0.954 (0.939–0.97)	< 0.001
**Ratio of PLNs**				
0%	Reference		Reference	
> 0%, ≤ 15%	0.528 (0.435–0.641)	< 0.001	0.522 (0.427–0.637)	< 0.001
> 15%, ≤ 30%	0.424 (0.334–0.538)	< 0.001	0.388 (0.299–0.503)	< 0.001
> 30%	0.476 (0.39–0.582)	< 0.001	0.383 (0.268–0.547)	< 0.001
Albumin	1.032 (1.008–1.058)	0.01	1.054 (1.027–1.082)	< 0.001
PLNs	0.959 (0.945–0.974)	< 0.001	1.019 (0.993–1.045)	0.162
**Age**				
≤ 45	Reference		Reference	
> 45, ≤ 55	0.918 (0.75–1.124)	0.408	0.982 (0.801–1.205)	0.863
> 55, ≤ 65	0.765 (0.609–0.96)	0.021	0.913 (0.722–1.156)	0.451
> 65	0.785 (0.607–1.017)	0.067	0.956 (0.731–1.25)	0.742
PBLC	1.117 (0.967–1.29)	0.132		
**Laterality**				
Left	Reference			
Right	0.953 (0.824–1.103)	0.519		
**Diabetes**				
No	Reference			
Yes	1.064 (0.855–1.325)	0.578		
**Type of intraoperative fluid**				
Crystalloid	Reference			
Crystalloid and colloid	0.904(0.768–1.064)	0.225		
**Use of painkillers after surgery**				
No	Reference			
Yes	1.17(0.829–1.652)	0.371		
DDCSD	0.95 (0.884–1.021)	0.162		

HR, hazard ratio; BMI, body mass index; DDCSD, drainage duration of chest wall suction drain; PBLC, peripheral blood lymphocyte count; PLNs, number of positive axillary lymph nodes; Ratio of PLNs, ratio of positive axillary lymph nodes; TLNs, number of total dissected axillary lymph nodes.

### Calibration and validation of the nomogram

**Figure 2:**
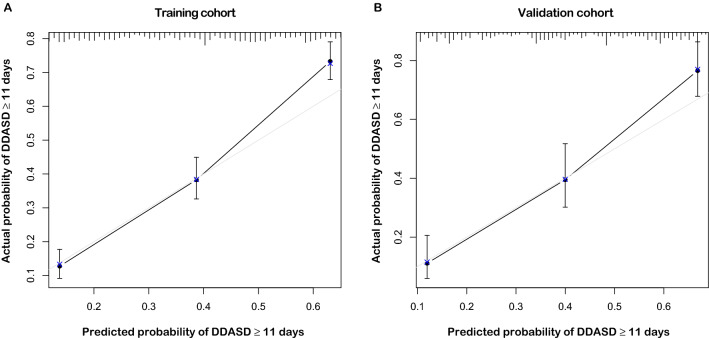
Calibration plots for checking the concordance between the nomogram‐predicted and actual probability of retaining the suction drain at the median DDASD in the (**A**) training cohort and (**B**) validation cohort. DDASD, drainage duration of axillary suction drain.

The calibration plots of the nomogram in the training and validation cohorts are presented in Fig. 2. A prediction probability of DDASD ≥ 11 days of more than 40% for the groups was considered slightly underestimated, while a probability of less than 40% was pretty accurate. On the whole, a good concordance existed between the predicted and actual probabilities.

**Figure 3:**
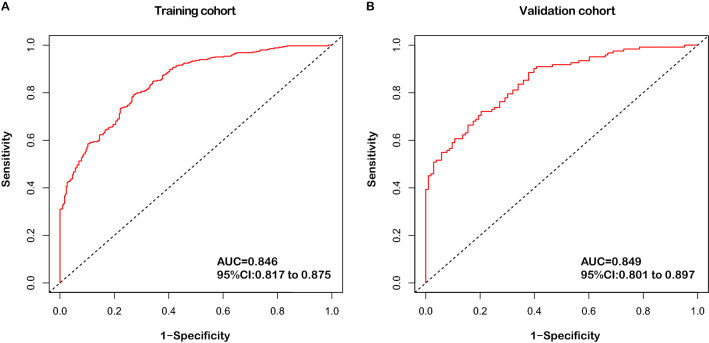
Receiver-operating characteristic curves (ROCs) for evaluating the performance of the nomogram-predicted probability of drainage duration of axillary suction drain ≥ 11 days in the training cohort and validation cohort. (**A**) The ROC curve in the training cohort indicates an area under the curve (AUC) of 0.846 (95% CI 0.817–0.875). (**B**) In the validation cohort, the ROC indicates an AUC of 0.849 (95% CI 0.801–0.897).

The C-index of the nomogram in the training and validation cohorts was 0.727 (95% CI 0.704–0.749) and 0.73 (95% CI 0.694–0.766), indicating good discrimination of this model. The ROC of the nomogram-predicted probability of DDASD ≥ 11 days is shown in Fig. 3. The AUC value of the ROC in the training cohort and validation cohort was 0.846 (95% CI 0.817–0.875) and 0.849 (95% CI 0.801–0.897), respectively, implying that the nomogram had good sensitivity and specificity in diagnosing patients with the DDASD equal to or more than 11 days.

## Discussion

Prevention of complications is an important part of the postoperative management of patients with BC. The use of a suction drain after MRM can significantly reduce the occurrence of seroma formation and infection^13,14^. However, prolonged drainage duration increases the risk of infection, as the drain itself may allow germs into the wound^15^. In addition, later removal of the suction drain also delays the opportunity for postoperative functional exercise, leading to a series of consequences, such as arm lymphedema, restriction of the shoulder movement, and self-care disorders^16^. Therefore, appropriate drainage duration is vital for the postoperative rehabilitation of patients with BC.

Some previous studies concluded that the suction drain should be removed when the drainage volume is 20–50 mL or less in 24 h^17–19^. In this study, the criterion for drain removal was total drain output of 20 mL or less daily for 2 days starting from postoperative day 3, which not only reduced the occurrence of postoperative subcutaneous effusion but also did not prolong the drainage duration too much. The median DDASD in this study was 11 days. Although it was longer than that reported in previous studies^18^, most patients accepted it. The DDASD exceeded 21 or even 30 days in a few patients. Hence, it was presumed that some larger lymphatic vessels might not be reached by an ultrasonic scalpel during the surgery, or the suture ligating the lymphatic vessels might loosen, resulting in lymphorrhagia^20^. To avoid the subsequent bias, patients whose DDASD exceeded 21 days were excluded.

Multivariate Cox analysis was performed in the training cohort, revealing several independent predictors. A high BMI significantly correlated with an increased risk of prolonged drainage duration. An interpretation for this might be as follows: obese patients usually had longer surgical time, larger surgical wounds, and richer subcutaneous adipose tissue than ordinary patients, leading to graver adipose tissue liquefaction by the thermal injury during the surgery^21,22^. Moreover, the poor blood supply of subcutaneous adipose tissue in obese patients also delayed wound healing, which also caused adipose tissue liquefaction, necrosis, and subcutaneous fluid after the operation, thus prolonging the duration of drainage^23^. The present study found that a longer drainage duration was significantly associated with the dissection of more lymph nodes, which was consistent with previous findings^24,25^. Some studies found that the drainage fluid produced during the early postoperative period of MRM was produced mainly by the leakage of blood vessels and lymphatic vessels destroyed during the surgery^17^. Gong et al. reported that the ligation of all lymphatic vessels connecting to the dissected lymph node specimens significantly reduced the postoperative drainage volume in patients who underwent MRM compared with the use of electrocautery or scalpel^26^. The ratio of PLNs was found to be an important predictor of drainage duration in this study. Petrek et al. shared a similar view on this^27^. A previous study showed that a large number of new lymphatic vessels were produced in the body due to the obstruction of lymphatic circulation after axillary lymph node metastasis^28^. Besides, excessive dissection of axillary lymph nodes and the destruction of more blood vessels and lymphatic vessels might also lead to an increase in postoperative drainage volume^27^. Hypertension has been shown as a significant predictor of drainage duration in many previous studies^6–8,10^. The fragility of capillaries and lymphatics were found to be increased in hypertensive patients, causing more postoperative bleeding and wound ooze. Meanwhile, a long-term history of hypertension was more likely to cause sodium-water retention, which might inhibit lymphatic circulation, thus increasing the risk of wound ooze and prolonging the drainage duration^29^. However, the relationship between the serum albumin level and postoperative drainage duration could not be explained. It was speculated that a low serum albumin level caused a decrease in plasma colloid osmotic pressure, thus increasing the volume of postoperative drainage fluid under the stimulation of vessel injury caused by surgery. Besides, a low serum albumin level might decrease the resistance to infection, thus prolonging the healing time of wounds and increasing the postoperative drainage volume.

The predictive model constructed in this study can help clinicians in clinical work. Using this model, clinicians can predict the estimated postoperative drainage duration and keep patients informed about the possible hospitalization days, eliminating their concerns to some extent. Clinicians can also identify patients with high probabilities of retaining the suction drain and take early intervention measures such as albumin infusion for patients with low serum albumin level. Moreover, patients with prolonged predicted drainage duration can be discharged home with the axillary suction drain when they recover well after surgery, which can decrease hospitalization days and costs of patients.

This study had some limitations. First, chemotherapy and the duration of elastic bandage use might influence postoperative drainage duration^13,24^; the present study had insufficient data on this, leading to a bias. Second, the participants in this study were from a single center in China, and therefore the predictive model might not be applicable to other centers. Furthermore, inevitable selection bias might have affected the conclusions due to the retrospective design of the study.

This study is novel in developing a nomogram for predicting drainage duration in patients with BC undergoing MRM. Undoubtedly, the nomogram can provide information for the postoperative drainage duration management of these patients. In addition, the sample size of this study was much larger than that of previous studies, increasing the statistical power and made the results more plausible.

## Conclusions

The study found out that BMI, serum albumin level, hypertension, TLNs, and ratio of PLNs were independent predictors of drainage duration in patients undergoing MRM. Based on this, a nomogram was constructed to predict the median postoperative drainage duration. The nomogram could predict the probability of retaining the suction drain during drainage, thus assisting clinicians in providing better and individualized postoperative management for patients with BC.

## Data Availability

The data that support the findings of this study are available from the corresponding author upon reasonable request.
